# Assessing radiofrequency safety of active implants by measuring induced radiofrequency currents using MRI

**DOI:** 10.1002/mrm.70084

**Published:** 2025-09-13

**Authors:** Chiara Hartmann, Mélina Bouldi, Jan M. Warnking

**Affiliations:** ^1^ U1216, Grenoble Institut Neurosciences Univ. Grenoble Alpes, Inserm Grenoble 38000 France; ^2^ KF‐EMV BAKOM Biel Switzerland

**Keywords:** active implantable medical device (AIMD), deep brain stimulation (DBS), MR safety, radiofrequency current, RF heating

## Abstract

**Purpose:**

During MRI in the presence of active wire‐like implants, such as deep brain stimulation leads, there is a risk of thermal lesions in tissues adjacent to implant contacts due to radiofrequency currents induced in the wire. Currently, there is no established method to evaluate the radiofrequency (RF) safety of an implant in situ, due to complex interactions between the implant and the electric field inside the patient during MRI. This article presents a method to quantify the RF current in an implant using MRI acquisitions at very low SAR.

**Theory and Methods:**

To measure RF current in situ, a modified B1‐mapping sequence is proposed to image the associated perturbation of the $B_{1}^{+}$ field. A forward signal model links the RF current intensity to the MRI signal and is used to fit the RF current from acquired data. Electromagnetic simulations and experiments on a homogeneous phantom are presented for simplified and real implant wires to validate the method.

**Results:**

The presented model can correctly reconstruct RF current amplitudes from field maps obtained with detailed electromagnetic simulations, with a normalized RMS error of 4.7%. Phantom experiments show a good linearity between the square of the current measured by MRI and temperature increase ($R^{2}>0.91$), demonstrating that the RF current measurements quantitatively represent the effective heating.

**Conclusion:**

A method has been developed to quantify the RF current in situ from MRI signals. This method enables to predict the individual heating risk for other MRI sequences performed in the same scanning session.

## 1 INTRODUCTION

MRI is considered particularly safe, since there are no known undesirable long‐term health effects of exposure to the electromagnetic fields used in MRI.^1^, ^2^ However, in the presence of medical devices, there are risks for the patient due to the interaction between implants and MRI electromagnetic fields.^3^ The present work focuses on the risk of radio‐frequency‐induced heating of active implantable medical devices (AIMDs), and in particular wire‐like implant components such as pacemaker or deep brain stimulation (DBS) leads. MRI exams in the presence of such devices can induce thermal lesions in tissues adjacent to the electrodes.^4^, ^5^

**FIGURE 1:**
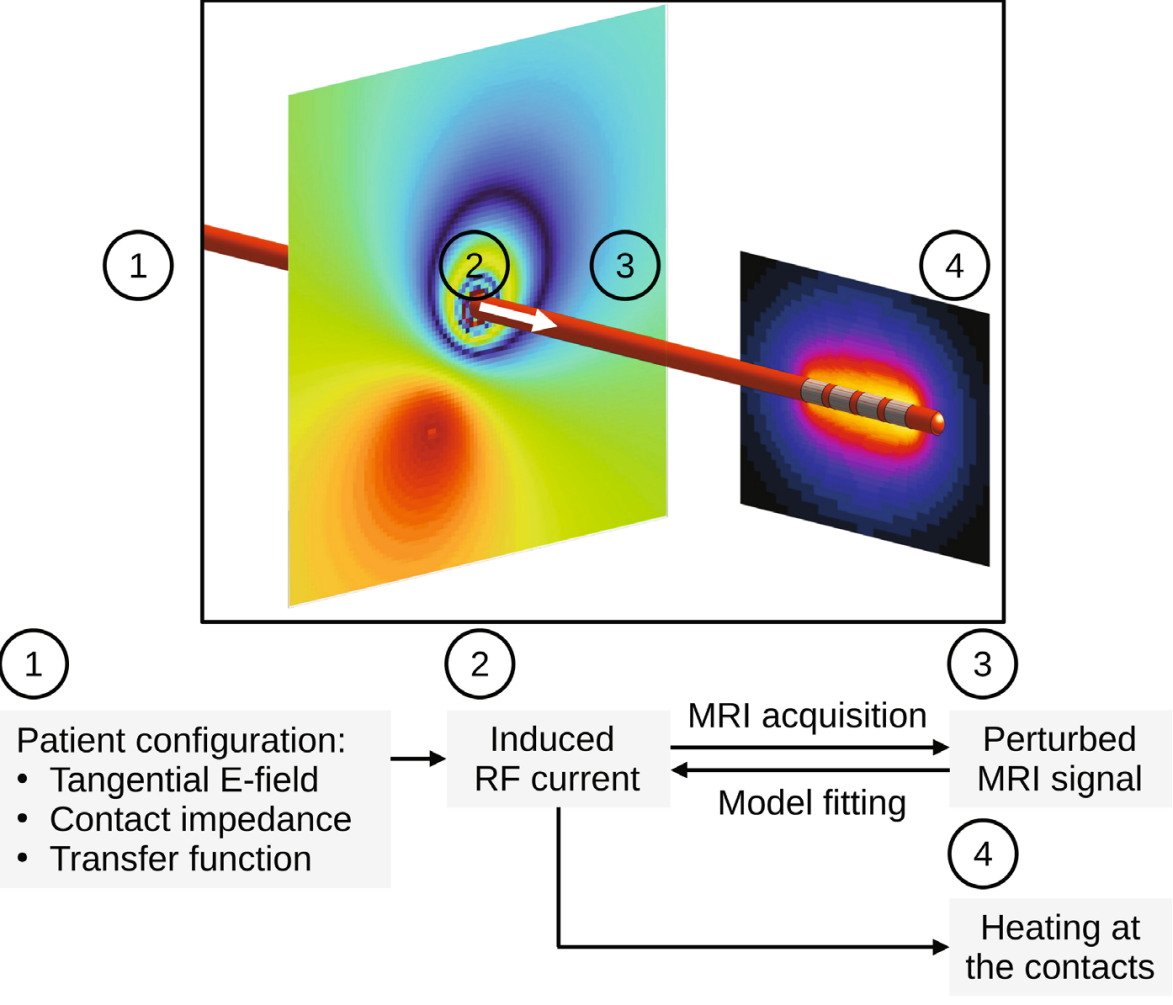
Principle of the RF heating prediction based on the MRI measurement of RF current close to the tip of a wire‐like implant. Complex and unknown configuration‐dependent interactions between MRI, patient, and implant (1) lead to an RF current close to the wire tip (2). This RF current perturbs the signal of a tailored MRI sequence (3) and can be inferred from the MRI signal. The RF current generates a scattered E‐field at the lead contacts, provoking heating in adjacent tissues (4). For a known implant geometry, this heating can be predicted from the measured RF current.

Radio frequency (RF) heating is caused by the RF electric field inside the patient, which is generated concomitantly with the RF magnetic field $B_{1}^{+}$ of the MRI. In the presence of a wire‐like implant, this background electric field induces an RF current in the lead, generating a scattered electric field, potentially leading to strong local heating in the tissues surrounding the lead tip (Figure 1).^6^, ^7^ The amplitude of the scattered field depends on the lead path and the distribution of the background E‐fields, both of which are highly variable across patients. To avoid RF heating in clinical practice, implantable medical devices undergo safety assessment based on electromagnetic simulations and in vitro studies in order to determine exposure conditions that are safe even for the worst case heating among all patients carrying the same implant.^8^, ^9^, ^10^, ^11^

Worst case limits are overly restrictive for many patients, and several studies have reported use of MRI without adverse events above the manufacturer's limits.^12^, ^13^, ^14^ When imaging protocols exceed these limits, clinicians need to decide if the risks of the MRI exam are balanced by its expected benefits,^15^ which is a difficult decision if no other information on the safety of the individual patient's configuration is available. More critically, patients may be totally refused from access to MRI in the case of nonstandard configurations of the implant, such as abandoned or damaged leads.^16^, ^17^

A method to reliably quantify the individual risk of RF heating in the presence of an AIMD for a patient at the beginning of an MRI exam could enable to detect both unexpected risks as well as safe configurations that may currently lead to refusal of access to MRI for lack of sufficient information. Quantifying the individual risk notably for the case of DBS leads may also help MR research on the neuromodulation induced by DBS, which is still not fully understood.^7^

RF currents in wire‐like implants produce a visible $B_{1}^{+}$‐artifact in MRI,^18^ and the potential to predict heating by using MRI signals to assess the RF current at the implant tip has long been recognized.^19^ Different methods to achieve this have been proposed: The current on a wire parallel to $B_{0}$ can be inferred from the $B_{1}^{+}$‐profile extracted from magnitude $B_{1}$‐maps along a circle around a wire.^20^ A high‐SNR, phase‐based method based on fast gradient‐echo acquisitions was shown to quantitatively predict RF heating, but requires a reciprocity relation between $B_{1}^{+}$ and $B_{1}^{-}$, mandating use of a whole‐body Tx/Rx coil.^21^ More simply, RF current amplitude in a wire can be calculated from the distance between the wire and the unique null of the total transmit field, assuming the background $B_{1}^{+}$ is constant so it can be measured in a slice not perturbed by the implant.^22^ All of these methods model the magnetic field provoked by the wire current quasi‐statically using Ampère's circuital law solved for a constant current on a straight wire. The effects of field propagation and nonconstant current along the wire can also be modeled,^23^ but fitting a complex 3D model over a large spatial scale seems impractical in vivo in the presence of signal heterogeneities.

In the present work, proof of concept for a method to accurately quantify the RF current and to predict RF heating induced in a wire‐like implant during MRI is provided, improving on previous work in several areas. A short, low‐SAR sequence optimized to capture a high dynamic range of $B_{1}^{+}$ values and robust to $T_{1}$ and $B_{1}^{-}$ heterogeneity is acquired in a single slice. The MRI signals are modeled directly as a function of the RF current amplitude in the implant, expanding on previously described models to allow for arbitrary wire angulation with respect to $B_{0}$.^24^ RF current is quantified by fitting data over a 2D patch around the wire, reducing the number of manual processing steps and making maximal use of the available information to optimize the current measurement precision. The area of MRI data used is kept small to ensure validity of the model and reduce sensitivity to field heterogeneities in vivo.

The theoretical basis of the workflow and its experimental validation is presented. The model of the $B_{1}^{+}$ around a wire was validated using 3D full‐wave EM simulations, and the capacity of the RF current measured to predict RF heating was validated in phantom experiments.

## 2 THEORY

### 2.1 Relationship between the RF current in the wire and RF heating

RF heating of tissues at the contacts of wire‐like implants is generated by the scattered electric field, which is strongest close to the small contact surfaces of the electrodes. The corresponding SAR distribution can be separated into a fixed spatial distribution, corresponding to a unit excitation amplitude and depending only on the electrode geometry, and a scalar amplitude factor depending on the lead configuration in the patient, lead properties and exposure conditions. In the transfer function approach, the amplitude factor is determined from the fixed transfer function of the wire and the tangential component of the background E‐field along its path, which is considered to be the only variable depending on lead configuration and exposure conditions.^9^ A similar result is obtained from a physically very different approach using the reciprocity principle, expressing the scale factor as the amplitude of a dipolar RF current source at the tip of the implant and the transfer function as the corresponding current distribution along the wire.^25^

Given the spatial distribution of the scattered electric field $\hat{E}\left(\vec{r}\right)$, resulting from a unit RF excitation, the corresponding normalized SAR can be calculated at positions $\vec{r}$:

(1) $$\hat{SAR}\left(\vec{r}\right)=\frac{\sigma \left(\vec{r}\right)}{2\rho \left(\vec{r}\right)}\hat{E}\left(\vec{r}\right){\hat{E}}^{*}\left(\vec{r}\right),$$

where $\sigma \left(\vec{r}\right)$ and $\rho \left(\vec{r}\right)$ are the electric conductivity and mass density of the surrounding medium. Importantly, the normalized SAR depends only in overall amplitude, but not in its spatial distribution, on the definition of the unit RF excitation, which could be unit input power to an RF coil, unit tangential E‐field along the implant or unit RF current at the wire tip. Given an actual RF current amplitude $I_{\text{tip}}$ at the wire tip in a given exposure condition and the RF current ${\hat{I}}_{\text{tip}}$ provoked by the unit RF excitation, the actual power deposition can be calculated by scaling the normalized SAR distribution by a factor ${\left|I_{\text{tip}}⁄{\hat{I}}_{\text{tip}}\right|}^{2}$.

This is equivalent to the RF safety evaluation according to ISO/TS 10974 clause 8 Tier 3, where the scale factor is derived from the transfer function of the implant and the background electric field derived from simulations.^8^ That same technical specification also provides standard procedures to determine $\hat{SAR}\left(\vec{r}\right)$, $\hat{dT}\left(\vec{r}\right)$ or equivalent RF power deposition data. Any metric that allows to derive exposure limits, such as total deposited power, peak SAR, 1‐g average SAR, or peak temperature increases after a given exposure duration, can be used. For the purpose of this work, we use local temperature increase at a fixed point $\vec{r}$ close to the temperature hot spot. The normalized distribution of RF heating $\hat{\Delta T}\left(\vec{r},t\right)$ at any time t after the start of the unit RF exposure can be calculated from $\hat{SAR}\left(\vec{r}\right)$ based on thermal simulations neglecting the effects of heat transfer due to boundary conditions and perfusion. This is valid in an inert phantom for an exposure duration on the order of minutes, and since perfusion has a cooling effect it is also routine practice for in vivo applications since it is a conservative approximation, introducing an additional safety factor in the RF heating estimated.

The normalized heating distribution obtained can then be simply scaled to derive the heating for an actual RF current amplitude $I_{\text{tip}}$ at the wire tip in a given exposure condition:

(2) $$\begin{array}{ll} dT\left(\vec{r},t\right) & =\hat{dT}\left(\vec{r},t\right)\cdot {\left|\frac{I_{\text{tip}}}{{\hat{I}}_{\text{tip}}}\right|}^{2}\\ & =c_{\text{implant}}\left(\vec{r},t\right){\left|I_{\text{tip}}\right|}^{2}, \end{array}$$

where $c_{\text{implant}}$ is an implant property that is independent of exposure conditions.

An accurate measurement of $I_{\text{tip}}$ thus summarizes the vast majority of the patient‐ and scanner‐dependent effects of RF heating due to the implant: the actual tangential E‐field generated by the MRI inside the patient's body over the lead length including proximity effects in the case of multiple leads, termination impedance of the lead at both ends, and the actual transfer function of the implant in the surrounding tissue including the effect of heterogeneous electric tissue properties. As a result, only the electric and thermal properties of the tissue at the lead tip are required for an accurate prediction of RF heating.

The proposed workflow for the use of an RF current measurement at the beginning of a scanning session to limit RF exposure to safe levels is as follows: Given the MRI‐derived RF current inside an implant lead, $I_{\text{MRI}}$, the nominal $B_{\text{1}\text{,}\text{rms}}^{\text{seq}}$ of the current measurement MRI sequence, and the constant in Equation (2) for the implant, $c_{\text{implant}}$, a maximum allowable nominal $B_{\text{1}\text{,}\text{rms}}^{\text{thresh}}$, leading to a threshold temperature increase of $dT_{\text{thresh}}$ can be determined:

(3) $$B_{\text{1}\text{,}\text{rms}}^{\text{thresh}}=B_{\text{1}\text{,}\text{rms}}^{\text{seq}}\sqrt{\frac{dT_{\text{thresh}}}{c_{\text{implant}}I_{\text{MRI}}^{2}}}.$$

This assumes that $I_{\text{MRI}}$ accurately measures $I_{\text{tip}}$, or more precisely, that the ratio $I_{\text{MRI}}⁄{\hat{I}}_{\text{MRI}}$ is the same as $I_{\text{tip}}⁄{\hat{I}}_{\text{tip}}$, where $I_{\text{MRI}}$ is the wire current in the MRI acquisition slice at an electrically short distance of a few centimeters from the tip and ${\hat{I}}_{\text{MRI}}$ is the corresponding current under unit exposure conditions. Previous work has shown that $I_{\text{MRI}}$ measured using a different method at 2.5 cm from an implant tip accurately predicts scattered SAR.^21^ Here we further validate this hypothesis experimentally for a range of positions of the MRI acquisition slice.

### 2.2 Model of the $B_{1}^{+}$ around a straight wire

The magnitude of the linearly polarized $B_{1}$‐field in an axial plane (perpendicular to $B_{0}$), generated by a time‐varying RF current I(t) through an infinite straight wire crossing the origin of the plane and placed at an angle $\xi_{j}$ to $B_{0}$, with an azimuthal angle $\theta_{j}$ in the axial plane, is:

(4) $$\left|{\vec{B}}_{1,j}(r,\theta_{r},t)\right|=\frac{\mu_{0}\mu_{r}I\left(t\right)\cos\xi_{j}}{2\pi r\left(1-\sin^{2}\xi_{j}\cos^{2}\left(\theta_{r}-\theta_{j}\right)\right)},$$

with r and $\theta_{r}$ the cylindrical coordinates in the axial plane, $\mu_{0}$ the permeability of free space, and $\mu_{r}$ the relative permeability of the medium surrounding the wire. This equation extends previously published work^20^ to the case of a wire of arbitrary angulation with respect to $B_{0}$. Its validity for implant leads with more complex internal structure, such as leads with multiple helical conductors, is further discussed in the supporting information.

The total transmit field $B_{1}^{+}$ is the sum of the background $B_{1}^{+}$ field generated by the RF transmit coil ($B_{1,b}$) and the left circularly polarized component of $B_{1,j}$. Substituting the more general expression of Equation (4) in the development presented in^20^ for the case of a wire parallel to $B_{0}$, the magnitude $\left|B_{1}^{+}\left(r,\theta_{r}\right)\right|$ of the total $B_{1}^{+}$ field as a function of position can be calculated,^24^ yielding Equation (5), with $\phi_{b}$ the phase of $B_{1,b}$, $\phi_{j}$ the phase of the RF current in the wire and $\phi_{j}^{\prime }=\phi_{j}-\phi_{b}$.

(5) $$\begin{array}{ll} & \left|B_{1}^{+}\left(r,\theta_{r}\right)\right|=B_{1,b}\sqrt{\begin{array}{cc} & 1+{\left(\frac{\mu_{0}\mu_{r}I\cos\xi_{j}}{4\pi rB_{1,b}\left(1-\sin^{2}\xi_{j}\cos^{2}\left(\theta_{r}-\theta_{j}\right)\right)}\right)}^{2}\\ & -\frac{\mu_{0}\mu_{r}I\cos\xi_{j}\sin\left(\theta_{r}-\phi_{j}^{\prime }\right)}{2\pi rB_{1,b}\left(1-\sin^{2}\xi_{j}\cos^{2}\left(\theta_{r}-\theta_{j}\right)\right)} \end{array}} \end{array}$$

Expressing $B_{1}^{+}$‐fields relative to the nominal value $B_{\text{1}\text{,}\text{nom}}$ introduces the dimensionless scale factors $\lambda \left(r,\theta_{r}\right)=B_{1}^{+}\left(r,\theta_{r}\right)/B_{\text{1}\text{,}\text{nom}}$ and $\lambda_{b}=B_{1,b}/B_{\text{1,nom}}$. Recognizing that I is induced in the implant by the electromagnetic fields generated concomitantly with $B_{1,b}$, the ratio of $I\text{/}B_{\text{1}\text{,}\text{nom}}$ at any instant is identical to the ratio of their root‐mean‐square (RMS) values $I_{\text{rms}}/B_{\text{1}\text{,}\text{rms}}$, leading to Equation (6). The full mathematical derivation is presented in the supporting information. Note that a reported wire tip current I is always referenced and linearly related to the background $B_{\text{1}\text{,}\text{rms}}$.

(6) $$\begin{array}{ll} & \left|\lambda \left(r,\theta_{r}\right)\right|=\lambda_{b}\sqrt{\begin{array}{cc} & 1+{\left(\frac{\mu_{0}\mu_{r}I_{\text{rms}}\cos\xi_{j}}{4\pi r\lambda_{b}B_{\text{1}\text{,}\text{rms}}\left(1-\sin^{2}\xi_{j}\cos^{2}\left(\theta_{r}-\theta_{j}\right)\right)}\right)}^{2}\\ & -\frac{\mu_{0}\mu_{r}I_{\text{rms}}\cos\xi_{j}\sin\left(\theta_{r}-\phi_{j}^{\prime }\right)}{2\pi r\lambda_{b}B_{\text{1}\text{,}\text{rms}}\left(1-\sin^{2}\xi_{j}\cos^{2}\left(\theta_{r}-\theta_{j}\right)\right)} \end{array}} \end{array}$$

In Equation (6), $\lambda_{b}$ is considered constant in space close to the wire, but local gradients in $\lambda_{b}$ were included in the numerical implementation of the model. $B_{1,b}$ varies smoothly for a volume transmit RF coil at up to 128 MHz, such that a first order spatial model is reasonable over a region of a few centimeters considered here. Higher spatial orders could be included if necessary. Finally, the position of the wire within the analyzed patch of MRI data was included as a fitted model variable to reduce manual interventions. All model parameters present in the numeric implementation used here are listed in Table 1.

**TABLE 1:** Sample‐dependent parameters of the physical forward model of $B_{1}^{+}$.

Parameter	Description
I	RF current along the wire
$\phi_{j}$	Phase difference between I and $B_{1,b}^{+}$
$\lambda_{b}$	Background $B_{1,b}^{+}$ in units of $B_{\text{1}\text{,}\text{nom}}^{+}$
$\lambda_{b,dx}$	Gradient of $\lambda_{b}$ on the *x*‐axis
$\lambda_{b,dy}$	Gradient of $\lambda_{b}$ on the *y*‐axis
$\xi_{j}$	Angle between the wire and $B_{0}$
$\theta_{j}$	Azimuthal angle wire (asial plane)
$x_{0}$	Wire offset from the origin in the x‐direction
$y_{0}$	Wire offset from the origin in the y‐direction
$\phi_{b}$	Phase of the background $B_{1,b}^{+}$

### 2.3 Dual‐Angle High Dynamic Range Actual Flip Angle sequence (da‐hdrAFI)

**FIGURE 2:**
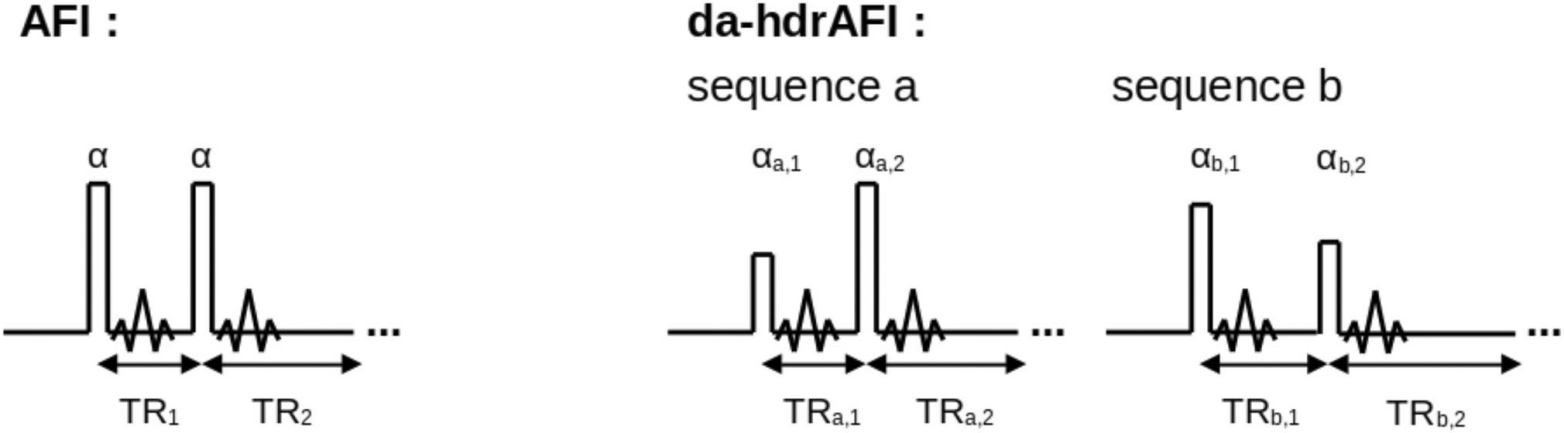
Schematic illustration of AFI and da‐hdrAFI sequences.

Measuring RF current from MRI signals using Equation (6) requires a sequence sensitive to $B_{1}^{+}$. Due to the $\frac{1}{r}$‐dependence of $B_{1,j}$, as well as constructive and destructive interference with $B_{1,b}$, the dynamic range of $B_{1}^{+}$‐values produced is much larger than typically encountered with volume‐transmit RF coils. MRI sequences with signal equations that are periodic in the local actual flip angle may fail to accurately represent the $B_{1}^{+}$ hotspot close to the wire. This is the case for the actual flip‐angle imaging (AFI) sequence, consisting of two interleaved gradient‐echo acquisitions with identical RF excitations but different TRs.^26^ To increase both sensitivity and dynamic range, we modified the AFI sequence by performing these two acquisitions using different excitation flip angles, and we acquired signals from two such dual‐angle AFI sequences with different acquisition parameters (sequences (a) and (b)), yielding four images in total (Figure 2). The sequence has been tested and has shown an increase of dynamic range of $B_{1}$‐mapping without loss of sensitivity in regions of low $B_{1}^{+}$.^27^

One da‐hdrAFI acquisition produces two sets of two signals. For each of the sequences, signal intensities can be calculated using the following analytical signal equations:

(7) $$\begin{array}{ll} S_{x,1} & =\frac{1-E_{x,2}+\left(1-E_{x,1}\right)E_{x,2}\cos\lambda \alpha_{x,2}}{1-E_{x,1}E_{x,2}\cos\lambda \alpha_{x,1}\cos\lambda \alpha_{x,2}}\sin\lambda \alpha_{x,1} \end{array}$$

(8) $$\begin{array}{ll} S_{x,2} & =\frac{1-E_{x,1}+\left(1-E_{x,2}\right)E_{x,1}\cos\lambda \alpha_{x,1}}{1-E_{x,1}E_{x,2}\cos\lambda \alpha_{x,1}\cos\lambda \alpha_{x,2}}\sin\lambda \alpha_{x,2}, \end{array}$$

with *x* indicating the sequence ((a) or (b)), $S_{x,1}$ the signal corresponding to the first excitation at a nominal flip angle of $\alpha_{x,1}$, $S_{x,2}$ the signal corresponding to the second excitation at nominal $\alpha_{x,2}$, λ the local relative $B_{1}^{+}$, $E_{x,1/2}=\exp\left(-\frac{TR_{x,1/2}}{T_{1}}\right)$, $T_{1}$ the local longitudinal relaxation time and $TR_{x,1/2}$ the repetition time associated with first/second excitation (see Table 2 for the list of sequence parameters). These equations are valid at any point in space. To eliminate the effects of local $B_{1}^{+}$ phase, $B_{1}^{-}$ amplitude and phase as well as $T_{2}^{*}$ relaxation, which are omitted from these equations, only magnitude data were used, normalized across the four signals for each voxel independently:

(9) $$\tilde{S_{x,i}}=\frac{\left|S_{x,i}\right|}{\sqrt{S_{a,1}^{2}+S_{a,2}^{2}+S_{b,1}^{2}+S_{b,2}^{2}}}.$$

This normalization also allows the da‐hdrAFI sequence to maintain the known robustness to $T_{1}$‐effects of the AFI sequence.

Intra‐voxel variations in $B_{1}^{-}$ are similar to those in $B_{1}^{+}$. To assess the impact of intra‐voxel $B_{1}^{-}$ heterogeneity on the voxel‐average signals, simulations either neglecting or accounting for intra‐voxel variations in $B_{1}^{+}$ amplitude and phase were performed using spatial oversampling. The median difference in normalized signals was 0.4% over a testing set of 10 000 model parameter vectors. The impact of intra‐voxel variations in $B_{1}^{-}$ is expected to be on the same order of magnitude, which was considered to be negligible.

**TABLE 2:** Sequence‐dependent parameters for the da‐hdrAFI sequence.

Parameters	Description
$\alpha_{a,1}$, $\alpha_{b,1}$	First nominal flip angles
$\alpha_{a,2}$, $\alpha_{b,2}$	Second nominal flip angles
$TR_{a,1}$, $TR_{b,1}$	First repetition times
$TR_{a,2}$, $TR_{b,2}$	Second repetition times

*Note*: Two sequences, (a) and (b), are acquired, for a total of 8 parameters.

## 3 METHODS

### 3.1 Forward MRI signal model

Throughout simulations and phantom experiments, sequence parameters used for the da‐hdrAFI sequence were $\alpha_{a,1}=45.2^{\circ }$, $\alpha_{a,2}=69.3^{\circ }$, $TR_{a,1}=23.7\;\text{ms}$, $TR_{a,2}=61.5\;\text{ms}$ and $\alpha_{b,1}=21.9^{\circ }$, $\alpha_{b,2}=39.0^{\circ }$, $TR_{b,1}=26.5\;\text{ms}$ and $TR_{b,2}=188.5\;\text{ms}$. These parameters were optimized to maximize sensitivity for RF current measurement. The detailed optimization procedure is outside the scope of this paper.

Equations 6 to 9 were used to build a forward model linking the sample‐dependent parameters (containing the current amplitude, Table 1) to corresponding MRI signals. Intra‐voxel gradients in $B_{1}^{+}$, which are strong in the vicinity of the wire, were taken into account by dividing each imaging voxel into 3×3 in‐plane sub‐voxels. To account for slice‐profile effects without incurring the cost of full Bloch equation simulations, we followed a hybrid approach. Specifically, a dictionary containing the average of the MRI signals at 201 through‐plane positions as a function of the value of λ at the center of the slice was precalculated using excitation slice profiles from Bloch equation simulations. The values of λ at the sub‐voxel centers were calculated using Equation (6) and MRI signals for each sub‐voxel were read from the dictionary. Finally, signals were averaged within each voxel and normalized using Equation (9).

### 3.2 Fitting the RF current from MRI signals

RF current in the implant was reconstructed along with nine other sample‐dependent model parameters (Table 1) from MR image data in a small patch of 12×12 voxels (24×24 mm) around the wire. Since the da‐hdrAFI sequence produces four MRI signals per voxel, this lead to a total of 576 input signals. Numeric model inversion was performed using a global stochastic parameter optimization by differential evolution (DE),^28^ with a population of 61 sets of model parameters over 122 iterations. The optimization minimized the root‐mean‐square (RMS) difference between signals simulated using the forward model and experimental MRI signals, masking signals within a 4‐mm radius around the fitted wire position. Calculation time was a few seconds per patch on a standard desktop computer. All practical details of implementation are available in the github repository cited in the “Data availability statement”.

### 3.3 Electromagnetic simulations

**FIGURE 3:**
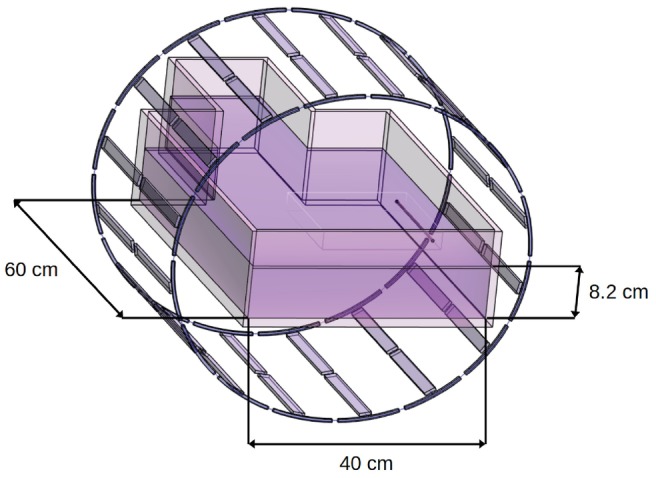
3D view of the model of the ASTM phantom filled with HEC gel and its dimensions in which a copper wire is placed and of the model of the elliptical band‐pass birdcage whole‐body transmit resonator.

In order to validate the $B_{1}^{+}$ model based on Ampère's law with respect to 3D full‐wave Maxwell‐equation simulations, harmonic EM simulations at 128 MHz were performed using Sim4Life (ZMT, Zürich, Switzerland) with a resonator model representing the 16‐rung elliptical birdcage whole‐body RF transmit coil used in our phantom experiments.^29^ The model included a phantom filled with hydroxyethylcellulose (HEC) gel, conforming to the ASTM (Advancing Standards Transforming Markets) standard F2182,^11^ and containing a straight 20‐cm copper wire of 3 mm diameter with insulation along the length and bare at both tips, placed either parallel to $B_{0}$ or tilted at 45° in a horizontal plane (Figure 3).

The simulated $B_{1}^{+}$‐field close to the wire was extracted and ground‐truth RF current in the wire was obtained using current sensors in the simulation, located in 41 axial planes at 5‐mm intervals along the wire. From the $B_{1}^{+}$‐field in each plane, MRI signals corresponding to the da‐hdrAFI sequence were calculated over a patch of 24mm×24mm centered on the wire using the forward MRI signal model and sub‐sampled to 12×12 voxels. RF current was then reconstructed from simulated MRI signals as described above and compared to the ground‐truth values extracted from the simulation.

To verify the validity of our model for the helical conductors inside a DBS electrode (theoretical discussion in Supporting Information, section 2), we performed a harmonic EM simulation with a high‐resolution model of a Medtronic DBS lead (model 3389‐40), in which an RF current was induced via a longitudinal RF E‐field at 128 MHz. We similarly compared the current fitted from simulated MRI signals to the simulated reference current.

### 3.4 Phantom experiments

MRI experiments were performed on an Achieva dStream 3.0T TX MRI (Philips, Netherlands) at the IRMaGe MRI facility (Grenoble, France) using an ASTM phantom containing HEC gel with a low‐frequency conductivity of 0.52 S/m. Due to the size of the phantom, the whole‐body RF coil was used for MRI signal reception. Two samples were examined independently, a straight 20‐cm copper wire of 3 mm diameter, insulated along the length with heat‐shrink tube and bare at both tips, and a DBS lead (Medtronic 3389‐40). Different configurations were tested per sample, each leading to different RF current amplitudes and heating.

For each configuration of the wire or lead, three MRI sequences were performed: a 3D FLASH sequence to document the geometric configuration, and sequences (a) and (b) of the da‐hdrAFI to measure RF current. The dual‐angle AFI sequence was developed in the Philips Pulse Programming Environment. A high‐intensity non‐selective off‐resonance RF pulse was added during the TR delay for each k‐space line acquired in sequence (a) to maximize the $B_{1,rms}$ of the sequence (2.29 µT, scanner‐reported SAR 2.6 W/kg) and thus generate strong heating. The absence of any effect of this heating pulse on acquired MR signals was verified in preliminary experiments.

Flip angles and repetition times for da‐hdrAFI were those specified for the forward MRI signal model. 19 axial 2‐mm slices with 2×2 mm in‐plane voxel size were acquired along the straight copper wire, spaced depending on the angulation of the wire to span the entire length (slice gap of 9 mm for the wire parallel to $B_{0}$) and 10 axial slices with a slice gap of 9 mm were acquired from the tip of the DBS lead. Acquisition duration was 25.8 s per slice in total for both sequences. The MRI‐derived RF current, $I_{\text{MRI}}$, was fitted in each slice from a patch of 12×12 voxels around the wire (576 signals from the 4 da‐hdrAFI images).

Thermometry data were acquired using fiber‐optic temperature probes (Photon Control, Canada) during a 5‐min baseline and the duration of the da‐hdrAFI sequence (a) (139 s for 19 slices). Probes were placed at both tips for the copper wire and the most distal electrode for the DBS lead. Data were baseline‐corrected and the relative scale in heating between different implant configurations was calculated from the thermometry time‐courses over the first 20 s of heating, the configuration with strongest heating serving as reference for all other configurations. The scaled peak temperature increase, $dT_{\text{thermo}}$, was recorded for each position.

**FIGURE 4:**
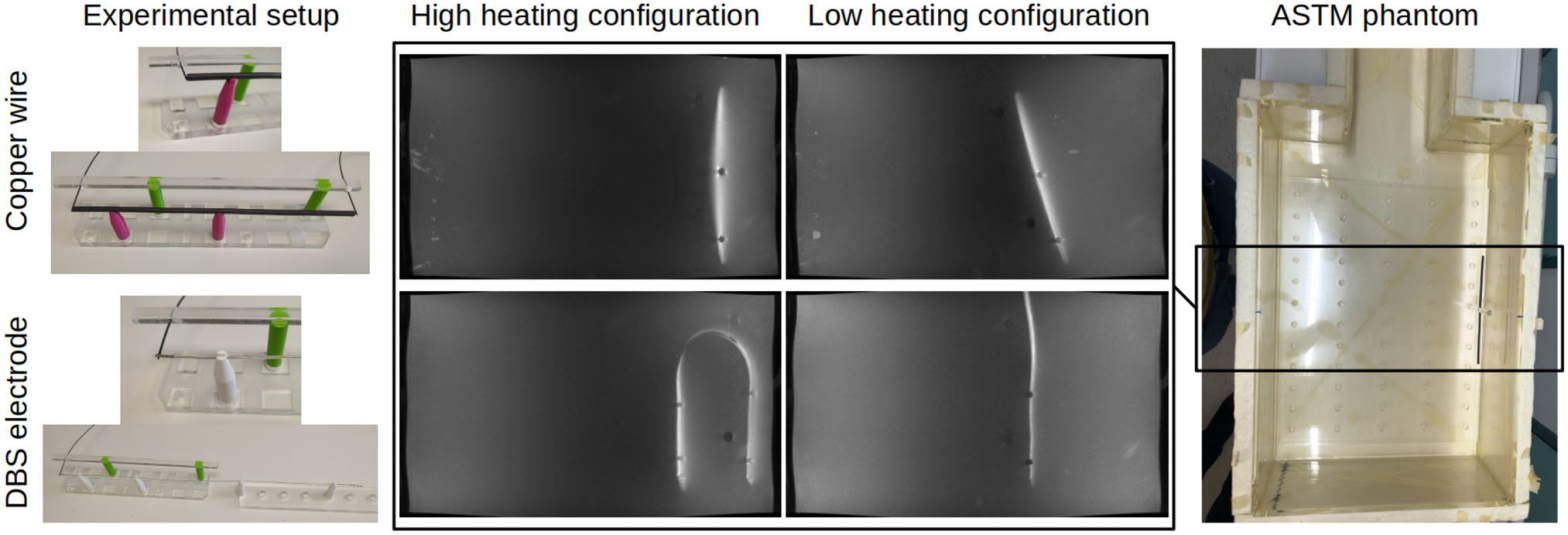
Experimental setups including temperature probes and test object configurations in the ASTM phantom shown by maximum‐intensity‐projections from the 3D FLASH acquisition (top row: straight copper wire, bottom row: DBS lead). The full set of configurations is shown in the supporting information (Figure S1).

The capacity of $I_{\text{MRI}}$ to predict RF heating was assessed by correlating $dT_{\text{thermo}}$ measurements with $I_{\text{MRI}}^{2}$ across implant configurations. A custom support assembly (Figure 4) was used to move the wire and probes together while keeping their relative positions fixed, and thus to maintain the linear relationship of Equation (2). Nine different translations and angulations of the straight wire, and eight different configurations of the DBS lead were tested, including translations of the distal part and different paths of the proximal part (Figure S1). The effect of the distance between MR acquisition slice and implant tip on the accuracy of the predicted RF heating was assessed by comparing correlations with observed heating for $I_{\text{MRI}}$‐values derived from each slice.

The precision of the method was further assessed for one chosen acquisition slice and all sample configurations based on absolute errors observed for three metrics:

1. Precision of current measurement: A ground‐truth wire current, $I_{\text{thermo}}$, was calculated from $dT_{\text{thermo}}$ using Equation (2) and compared to $I_{\text{MRI}}$.
2. Precision of heating prediction: The MRI‐predicted heating using Equation (2), $dT_{\text{MRI}}$, was compared to $dT_{\text{thermo}}$.
3. Precision of heating at $B_{\text{1}\text{,}\text{rms}}^{\text{thresh}}$: The maximum allowable $B_{\text{1}\text{,}\text{rms}}$ was determined from $I_{\text{MRI}}$ and Equation (3) for a target $dT_{\text{thresh}}=2\;K$. The threshold temperature increase predicted from thermometry, $dT_{\text{thermo}}\cdot {\left(B_{\text{1}\text{,}\text{rms}}^{\text{thresh}}/B_{\text{1}\text{,}\text{seq}}\right)}^{2}$, was compared to the target $dT_{\text{thresh}}$.

All comparisons require knowledge of the proportionality constant $c_{\text{implant}}$ of Equation (2), which was determined from a linear fit to a set of observed pairs of $dT_{\text{thermo}}$ and $I_{\text{MRI}}$ from several implant positions. To avoid bias, a leave‐one‐out approach was used, evaluating each pair of $I_{\text{MRI}}$ and $dT_{\text{thermo}}$ using a value of $c_{implant}$ fitted from the N‐1 remaining data pairs.

## 4 RESULTS

### 4.1 Electromagnetic simulations

**FIGURE 5:**
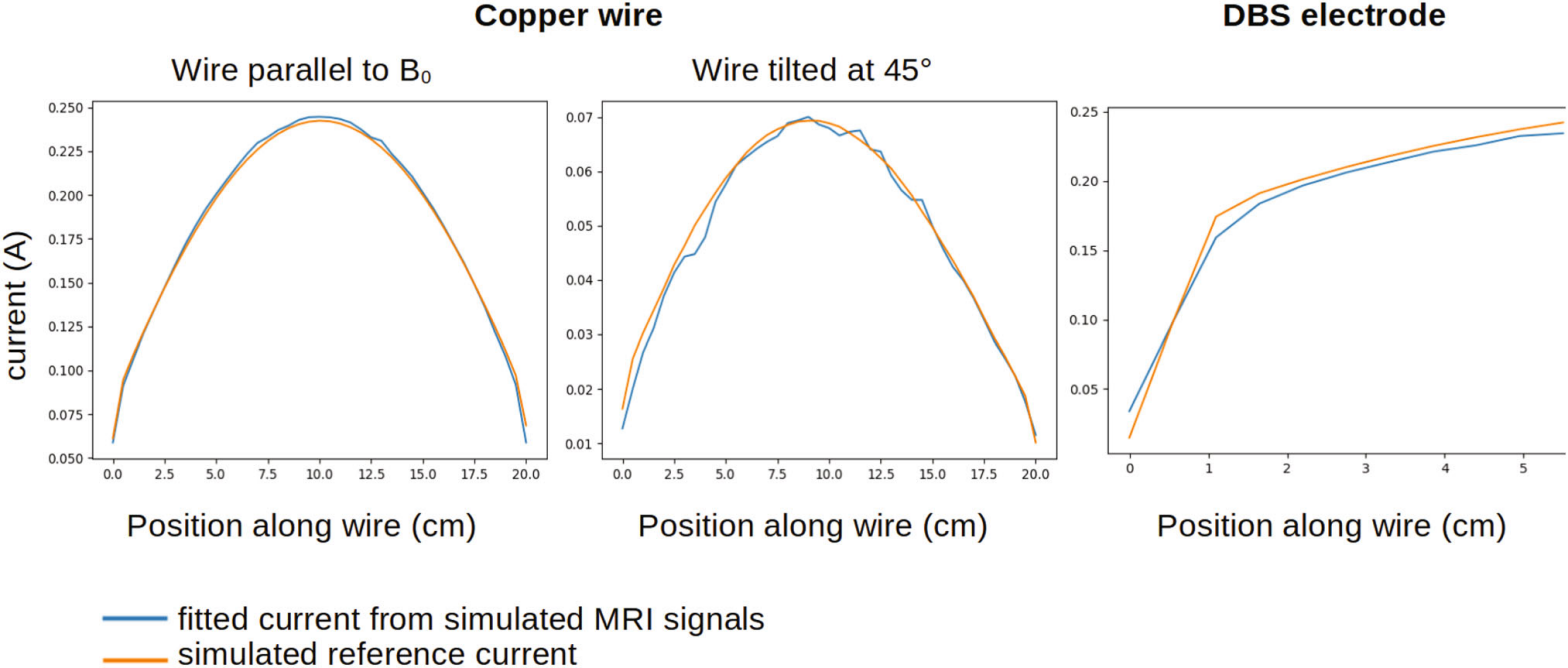
Simulated and fitted currents from electromagnetic simulations for the copper wire parallel to $B_{0}$ and tilted at 45° and the realistic model of a DBS electrode. For the copper wire, excitation was performed with the birdcage coil, the field being scaled to a $B_{\text{1}\text{,}\text{rms}}$ of 2.2 μT at the center of the phantom. For the DBS electrode, the excitation was performed with a tangential E‐field, scaled to elicit an RF current on the same order of magnitude as observed in the phantom experiment.

The median of absolute values of the relative difference between fitted and simulated MRI signals was 0.19% (wire parallel to $B_{0}$), 0.18% (wire tilted at 45°) and 0.33% (DBS electrode). Figure 5 shows the RF current fitted from synthetic MRI data along with the ground truth current amplitudes from EM simulations for both wire orientations and for the initial portion of the DBS electrode. Mean absolute error of fitted current with respect to the simulated one was 2.3 mA (wire parallel to $B_{0}$), 1.4 mA (wire tilted at 45°) and 7.3 mA (DBS electrode) and the normalized RMS error was 1.5% for the wire parallel to $B_{0}$, 4.0% for the wire tilted at 45° and 4.7% for the DBS electrode.

### 4.2 Phantom experiments

**FIGURE 6:**
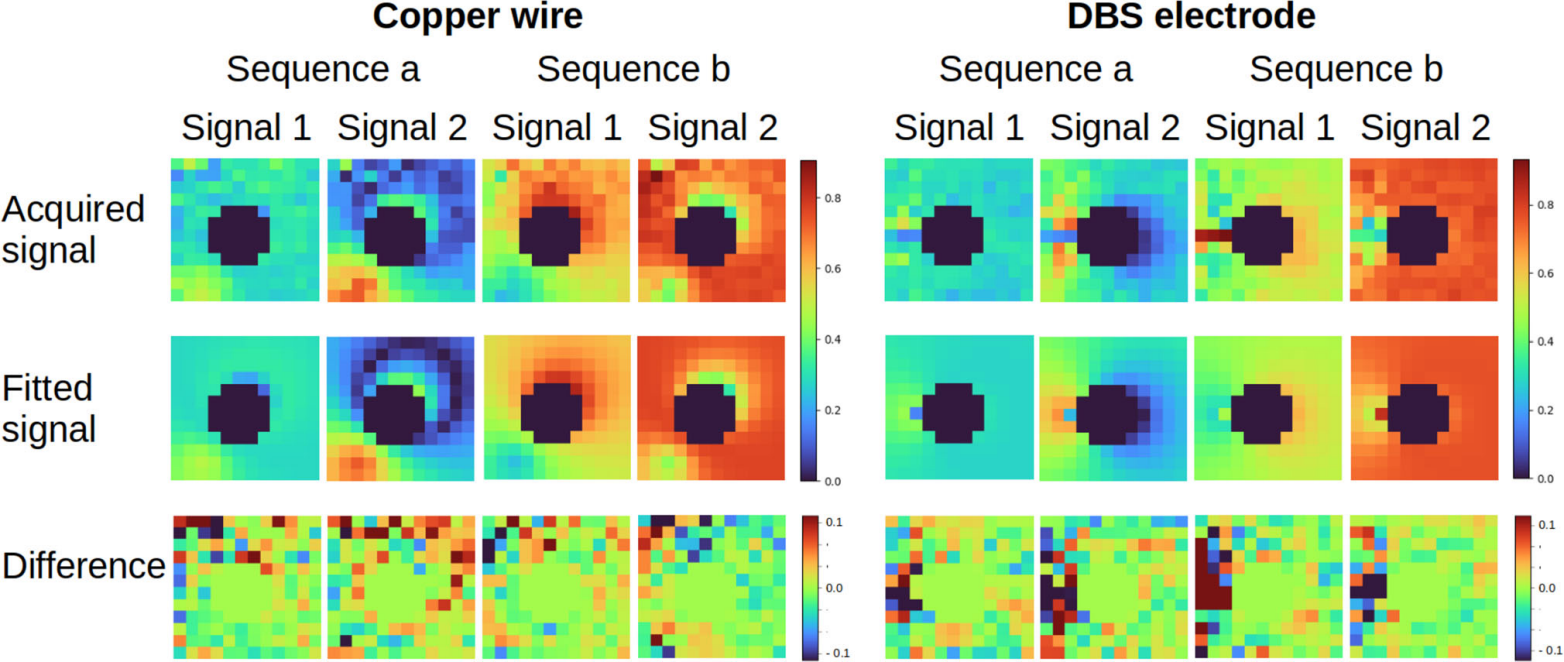
Examples of acquired and corresponding fitted signals in the slice at 33 mm from the tip of the copper wire (left) and the DBS electrode (right) for the configurations with highest heating (Position 1 for the straight wire and Position 2 for the DBS electrode, as shown in Figure S1).

All acquired data showed the presence of RF current on the wire or the DBS lead. The 3D FLASH sequence qualitatively shows the current amplitude profile along the wires by the hyperintensities in the maximum‐intensity‐projections in the coronal plane (Figure 4). Figure 6 shows an example of acquired and corresponding fitted da‐hdrAFI signals for one configuration and one slice for both copper wire and DBS lead. The median across voxels of the absolute values of the relative difference between acquired and fitted MRI signals from all implant configurations and all slices are between 3.9% and 4.9% (copper wire) and between 4.0% and 5.5% (DBS lead). The region of large signal deviations seen to the left of the DBS lead in Figure 6 are due to the presence of a support post in that slice.

**FIGURE 7:**
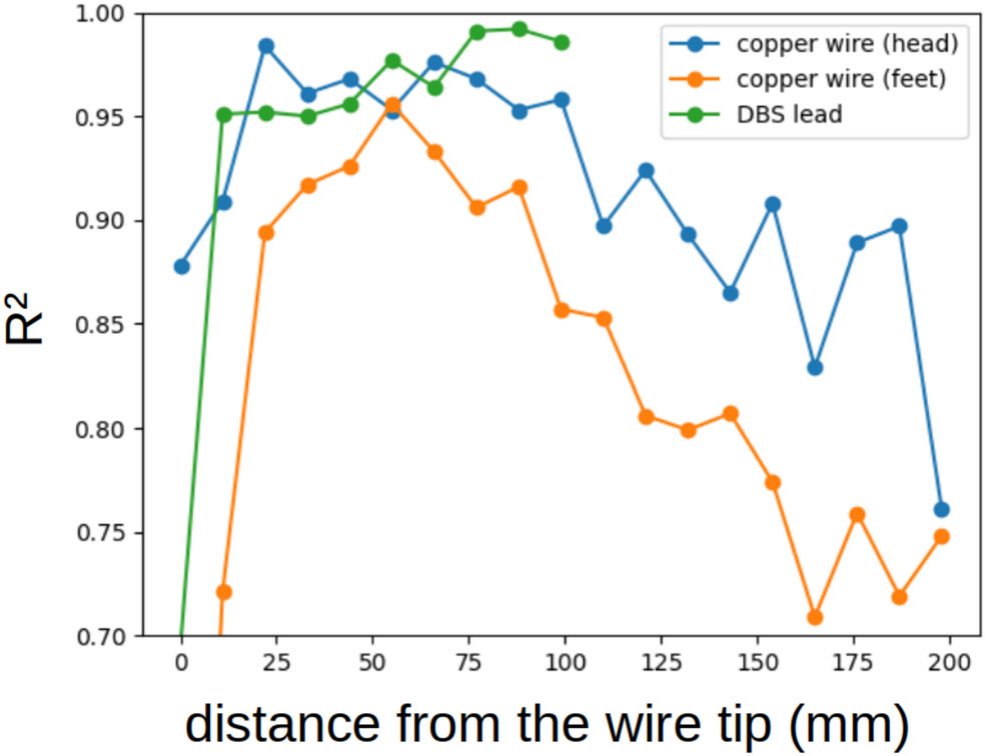
Coefficient of determination ($R^{2}$) for the linear fit between $dT_{\text{thermo}}$ and $I_{\text{MRI}}^{2}$ across implant positions, calculated independently for $I_{\text{MRI}}$ values derived from each acquired slice, as a function of the distance between the slice and the wire tip at which thermometry was performed.

RF currents measured via MRI correlated closely with heating measurements, coefficients of determination ($R^{2}$) approaching or exceeding 0.9 for all thermometry locations and all acquired slices between 22 mm and 88 mm from the wire tips where thermometry was performed (Figure 7). For further analysis of the precision of the RF current measurement, the slice located at 33 mm from the tips was chosen.

**FIGURE 8:**
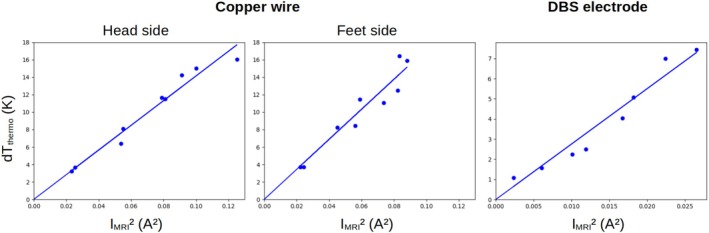
Comparison between the temperature increase measured with temperature probes at the implant tips and the square of the RF current measured by MRI in slices centered at a distance of 33 mm, for both tips of the straight wire and for the DBS electrode (dots). The solid lines indicate the least‐squares fit to the data for a strictly proportional relationship between RF current and temperature change (Equation 2).

Figure 8 shows the correlation between thermometry data and RF current measured by MRI from that slice, for the two wire tips and the DBS lead. The dots represent the measurements and the solid line the linear fit to all data points. The linear fits lead to $R^{2}$ values of 0.96 (copper wire head side), 0.92 (copper wire feet side) and 0.95 (DBS lead). The median (range) of the absolute error between $I_{\text{MRI}}$ and $I_{\text{thermo}}$ for the copper wire head side was 7 mA (1–24 mA), for the copper wire feet side 17 mA (2–26 mA) and for the DBS lead 9.5 mA (1–14 mA). The median (range) of the absolute error between $dT_{\text{thermo}}$ and $dT_{\text{MRI}}$ for the copper wire head side was 0.6 K (0.0–2.4 K), for the copper wire feet side 1.3 K (0.1–2.59 K) and for the DBS lead 0.5 K (0.1–1.1 K). Among configurations where heating of at least 2 K was possible on our MRI, the maximal deviation of heating at the calculated $B_{\text{1}\text{,}\text{rms}}^{\text{thresh}}$ with respect to the target $dT_{\text{thresh}}=2\;K$ was 0.33 K (copper wire head side), 0.37 K (copper wire feet side) and 0.5 K (DBS lead). Complete data are provided in Tables S1–S3.

## 5 DISCUSSION

A method to quantify RF current in an implant wire using MRI signals was developed and evaluated. The current amplitude was obtained by fitting a signal model to a small patch of MRI data from a custom sequence. This work builds on previous similar methods and aims to extend their validity, robustness, and ease of use. The model presented applies to any wire angulation except perpendicular to $B_{0}$, in contrast to previous methods limited to implants parallel to $B_{0}$.^20^ It was validated for angulations of up to 45° with respect to $B_{0}$ and accurately reconstructed RF currents from acquired and simulated MRI signals.

The signal model consists of two parts, first the total $B_{1}^{+}$ field in the vicinity of the wire, and second the MRI signals as a function of $B_{1}^{+}$. Both parts contain approximations that were validated independently.

The model of $B_{1}^{+}$ close to the wire uses Ampère's law, assuming an RF current I that is constant in amplitude and phase along a straight infinite wire. The validity of this model in the present context was checked against full‐wave 3D EM simulations of $B_{1}^{+}$ around a straight wire and a helical DBS lead. The RF current fitted from synthetic MRI signals matched the ground‐truth values to within 4.7% (Figure 5), validating the simplified model of the $B_{1}^{+}$ close to a wire, even for a more complex wire geometry. Importantly, fitted currents matched ground truth values well even close to the tips where the hypothesis of an infinite current is least respected. For implants that are highly curved close to the implant tip, such as cochlear implants, the present model may not be suitable however.

The hypothesis of a spatially constant phase of $B_{1,b}$, $\phi_{b}$, over the size of the MRI data patch used ($24\times 24\;mm^{2}$) was considered reasonable for a homogeneous phantom at 3 T. This requires further validation in vivo depending on the anatomical region targeted. A spatial gradient of $\phi_{b}$ could be added to the model if necessary.

The MRI signal model was validated via phantom experiments. The forward model was able to accurately reproduce measured MRI data acquired around a solid copper wire and a DBS lead, proving that intra‐voxel heterogeneity of $B_{1}^{+}$ as well as slice profile effects are modeled well. $T_{1}$ heterogeneities were not present here, and while the sequence is expected to be robust to them, this still requires validation in a heterogeneous environment.

The final validation regards predicted heating. We observed a good prediction of heating from MRI‐measured currents for a wide range of acquisition slice positions between 22 mm and 88 mm from the implant tip (Figure 7). The lower value is also in line with previously reported findings.^21^ This leads us to conclude that a range of positions satisfies both the lower limit on tip‐to‐slice distance, from using Ampère's law, and the upper limit, to correctly represent $I_{\text{tip}}$. The conversion factor between RF current and heating does however depend on tip‐to‐slice distance, requiring repeatable slice placement.

The present method improves on previously presented work in several ways. Directly fitting RF current from MRI data avoids the need in other methods to calculate an intermediate $B_{1}^{+}$‐map,^20^ to determine a background $B_{1}^{+}$‐value from a different slice^22^ or to adjust a model over a large volume of the sample.^21^, ^23^ The fit requires few manual interventions except positioning an axial acquisition plane at a fixed distance from the implant tip and selecting the approximate position of the implant in the slice. Integrating data from a patch of voxels increases robustness of these operations, the exact wire position being among the fitted parameters.

The RF current measurement proved robust to the low SNR of MRI signals acquired here using the body RF‐coil for signal reception. This is likely due to the sensitivity of the da‐hdrAFI sequence and the signal analysis over many voxels. The precision when acquiring signals with a head receive coil is expected to be much better. Importantly, the method makes no assumption regarding $B_{1}^{-}$ and is thus compatible with any receive RF coil.

Experiments on the DBS lead were performed without connection to an implantable pulse generator (IPG). The presence of an IPG, like the changes in lead routing examined here, impacts heating at the distal electrodes via a change in RF current along the lead. MRI‐measured RF current is expected to remain a good predictor of RF heating, since the constant $c_{\text{implant}}$ of Equation (2) at the distal end is independent of proximal termination impedance.

Practical considerations may be a limitation for this method, since it aims to predict RF heating during a clinically useful acquisition sequence from an RF current measurement early in the scanning session. Significant changes in the RF E‐field around the implant during the session require repeating the RF current measurement. Potential causes for such changes are modifications of scanner or patient configuration, including severe patient motion, table movement during the session, modification of pTX parameters and of scanner RF power calibration. In this study, the RF transmit shim was fixed. Many of these causes can be ruled out by blocking changes in the relevant scanner parameters, which may however require low‐level control over the scanner.

Scanner‐reported SAR of the da‐hdrAFI sequences used here is 0.4 W/kg for sequence (a) ($B_{\text{1}\text{,}\text{rms}}=0.84\;\mathrm{\mu T}$) and 0.0 W/kg for sequence (b) ($B_{\text{1}\text{,}\text{rms}}=0.28\;\mathrm{\mu T}$). SAR should be further optimized to below 0.1 W/kg to perform the sequence safely for as many implants as possible. Preliminary experiments with other sequence parameters indicated that current measurement is possible over a large range of parameters. The duration of the da‐hdrAFI used here is 25.8 s for one slice, which is perfectly adapted to this kind of pre‐scan.

The method could be adapted to other $B_{1}$‐mapping sequences such as DREAM,^30^ which is faster, but may not be well suited for this application. First, single‐shot DREAM does not provide low enough voxel sizes over a large field‐of‐view, and multi‐shot DREAM in a single slice is slower than AFI. Second, the long readout may induce blurring, possibly affecting the capacity to accurately fit RF current. Third, the intrinsically lower dynamic range of DREAM is a disadvantage here. And finally, the measurement of the RF current only requires acquisition of a single 2D slice, provoking slice‐profile effects. Modeling them accurately for DREAM may require full simulation of Bloch equations during the fitting process, drastically increasing processing time.

Applying the proposed method in a patient to derive an individually tailored exposure limit requires knowledge of the calibration constant in Equation (2) for the type of implant(s) present. This is equally the case for the usual evaluation of RF safety performed for active implants^8^ or a workflow similar to ours proposed recently.^31^

From there, the RF current in the implant can be measured for an individual patient in a given scanner, RF coil and position. Subsequently, the maximal $B_{\text{1}\text{,}\text{rms}}^{\text{thresh}}$ the patient can be exposed to without exceeding a threshold temperature increase can be determined. The method is expected to work in abandoned and broken leads, unless the lead is damaged close to the tip or in case of heating near damage‐induced electrical contact between lead and tissue. If there are several wire tips present, the RF current measurement needs to be performed for each one. Proximal interactions between several implants are accounted for, but the impact of a second implant close to the extracted patch of MRI data used to measure RF current has not been evaluated. The present work was performed at a field strength of 3 T, but the same methods and principles are valid at a lower clinical field strength. SNR is expected to be sufficient at lower field when using local receive coils, given the precision observed here with a whole‐body RF coil. At higher field, especially above 7 T, adaptations may be necessary to fullfill the model hypotheses discussed above. More fundamentally, the absence of MRI safety testing of medical devices by their manufacturers at high field may preclude performing even the proposed low‐SAR prescan.

## 6 CONCLUSIONS

This article presents a method to measure the RF current in a wire‐like implant for an individual configuration in a patient by MRI. The method is based on a modified $B_{1}$‐mapping sequence and a model of the MRI signal as a function of sample dependent parameters including RF current amplitude, allowing to fit the current from MRI data acquired in a patch close to the wire. The analytical expression of the $B_{1}^{+}$ as a function of wire current was validated by full‐wave EM simulations. The ability of the method to quantitatively predict RF‐heating was validated by temperature measurements at the tip of a copper wire and a DBS lead. This method, combined with EM simulations to determine the calibration constant, may constitute a technique to derive an RF exposure limit individually tailored for a given patient, a given scanner setting and a given implant configuration, including abandoned or broken leads.

## CONFLICT OF INTEREST STATEMENT

The authors declare no potential conflict of interest.

## Supporting information




**Data S1.** Supplementary Information.

## Data Availability

The code used to fit the current from da‐hdrAFI signals is provided in the following github repository: https://github.com/nifm‐gin/RF_current_from_MRI.git (Release v1.0.0). Data from phantom experiments of this article are also available to test the code in the repository.
