# Supplementary material for: Assessing radiofrequency safety of active implants by measuring induced radiofrequency currents using MRI
Source: Magn Reson Med. 2025 Sep 13;95(2):868–80. doi: 10.1002/mrm.70084 (PMC12681316; doi:10.1002/mrm.70084)
Supplement: Supplementary file 1 — Data S1. Supplementary Information. [file MRM-95-868-s001.pdf]

1 | DERIVATION OF THE ANALYTICAL EXPRESSION OF B_1^+ AROUND A STRAIGHT WIRE

Let $\vec{j(t)} = I(t)\,\vec{e}_j$ be a radio-frequency alternating current of amplitude $I(t)$ along a straight wire with direction $\vec{e}_j$. We adopt a coordinate system in which the B_0-field of the the MRI is aligned with the z-axis and in which the wire passes through the origin of the coordinate system. The $\vec{B}$-field generated by this current as a function of the position $\vec{r}$ is circular around the wire and its amplitude is inversely proportional to the distance from the wire:

$$\vec{B}_j(\vec{r},t) = \frac{\mu_0\mu_r I(t)}{2\pi\left|\vec{r}-\vec{e}_j(\vec{e}_j\cdot\vec{r})\right|}\frac{\vec{e}_j\times\vec{r}}{\left|\vec{e}_j\times\vec{r}\right|} \tag{1}$$

$$= \frac{\mu_0\mu_r I(t)}{2\pi}\frac{\vec{e}_j\times\vec{r}}{\left|\vec{e}_j\times\vec{r}\right|^2} \tag{2}$$

where $\mu_0 = 4\pi\cdot 10^{-7} H/m$ is the permeability of free space (SI units) and μ_r is the relative permeability of the sample in which the wire is placed. We examine the B-field in the near-field approximation, which holds for locations much closer to the wire than the EM wavelength in the medium considered at the frequency of oscillation of the current $I(t)$.

The current vector forms an angle $\xi_j < \pi/2$ with the z-axis of the coordinate system, and its projection to the xy plane an azimuthal angle θ_j with the x-axis of the coordinate system:

$$\vec{e}_j = \begin{pmatrix}\sin\xi_j\cos\theta_j\\ \sin\xi_j\sin\theta_j\\ \cos\xi_j\end{pmatrix}. \tag{3}$$

The $\vec{B}_1$-field generated by the current in the wire is linearly polarized at each location in space and consists of the x- and y-components of the above $\vec{B}$ vector:

$$\vec{B}_{1,j} = \vec{B}_j\Big|_{xy} \tag{4}$$

We calculate expressions for observation of $\vec{B}_1$ in the x-y plane. Since for $\xi_j = \pi/2$ the $\vec{B}$-field generated by the wire is parallel to the z-axis of the magnet in the x-y plane, the corresponding $\vec{B}_1$-field vanishes and we exclude that case in the following calculations. Expressing the position $\vec{r}$ in the x-y plane in cylindrical coordinates, such that

$$\vec{r} = \begin{pmatrix}r\cos\theta_r\\ r\sin\theta_r\\ z_r\end{pmatrix}, \tag{5}$$

it can be shown that in the plane $z_r = 0$, and for $\xi_j \neq \pi/2$, Eq. 2 for the x and y components of $\vec{B}_1$ becomes

$$\vec{B}_{1,j}(\vec{r},t) = \frac{\mu_0\mu_r I(t)\cos\xi_j}{2\pi r\left(1-\sin^2\xi_j\cos^2(\theta_r-\theta_j)\right)}\begin{pmatrix}-\sin\theta_r\\ \cos\theta_r\end{pmatrix}. \tag{6}$$

As noted above, this B_1-field is linearly polarized. Writing the radio-frequencey current $I(t) = I\cos(\omega t+\phi_j)$, with a Larmor frequency ω and a phase offset ϕ_j, and adopting a complex notation for the xy-component of the B_1-field where $B_1 = \vec{B}_1\Big|_x + i\vec{B}_1\Big|_y$, we can decompose this field in a left circularly-polarized and a right circularly-polarized component:

$$B_{1,j}(r,\theta_r,t) = \frac{\mu_0\mu_r I\cos(\omega t+\phi_j)\cos\xi_j}{2\pi r\left(1-\sin^2\xi_j\cos^2(\theta_r-\theta_j)\right)}ie^{i\theta_r} \tag{7}$$

$$= \left|\vec{B}_{1,j}(r,\theta_r)\right|\frac{\left(e^{i(\omega t+\phi_j)}+e^{-i(\omega t+\phi_j)}\right)}{2}ie^{i\theta_r} \tag{8}$$

$$= \frac{\left|\vec{B}_{1,j}(r,\theta_r)\right|}{2}i\left(e^{i(\omega t+\phi_j+\theta_r)}+e^{-i(\omega t+\phi_j-\theta_r)}\right), \tag{9}$$

where

$$\left|\vec{B}_{1,j}(r,\theta_r)\right| = \frac{\mu_0\mu_r I\cos\xi_j}{2\pi r\left(1-\sin^2\xi_j\cos^2(\theta_r-\theta_j)\right)}. \tag{10}$$

The effective transmit field B_1^+ consists only of the left circularly-polarized component:

$$B_{1,j}^+(r,\theta_r,t) = i\frac{\left|\vec{B}_{1,j}(r,\theta_r)\right|}{2}e^{-i(\omega t+\phi_j-\theta_r)} \tag{11}$$

The background $\vec{B}_1^+$-field generated by the RF transmit coil is assumed constant in space over the distances considered here, and has a left circularly polarized component of

$$B_{1,b}^+(\vec{r},t) = B_{1,b}e^{-i(\omega t+\phi_b)}. \tag{12}$$

Since the current induced in the wire is provoked indirectly by the background B_1-field, via the concomitantly created E-field, any shift in ϕ_b due to the pulse sequence leads to an equivalent shift in ϕ_j. We can then decompose the phase of the RF current into the phase shift with respect to the background field ϕ_j', which is dependent on the geometry of the problem only, and possibly on the relative phase and amplitude factors between RF transmit coils in a parallel-transmit setup, and the phase of the background field: $\phi_j = \phi_j' + \phi_b$. The total effective transmit field close to the wire is then:

$$B_1^+(r,\theta_r,t) = B_{1,b}^+(r,\theta_r,t) + B_{1,j}^+(r,\theta_r,t) \tag{13}$$

$$= e^{-i(\omega t+\phi_b)}\left(B_{1,b} + i\frac{\left|\vec{B}_{1,j}(r,\theta_r)\right|}{2}e^{-i\left(\phi_j'-\theta_r\right)}\right). \tag{14}$$

The magnitude of the effective transmit field can then be calculated as shown in Eqs. 15 to 17. Writing this equation in full, we obtain Eq. 18, yielding identical results to[1] for the case of $\xi_j = 0$. The phase offset of the B_1^+-field with respect to the theoretical phase of $-\omega t$ is given in Eqs. 19 and 20.

$$\left|B_1^+(r,\theta_r)\right| = \left|B_{1,b} + i\frac{\left|\vec{B}_{1,j}(r,\theta_r)\right|}{2}e^{-i(\phi_j'-\theta_r)}\right| \tag{15}$$

$$= \left\{\left(B_{1,b} + \frac{\left|\vec{B}_{1,j}(r,\theta_r)\right|}{2}\sin\left(\phi_j'-\theta_r\right)\right)^2 + \left(\frac{\left|\vec{B}_{1,j}(r,\theta_r)\right|}{2}\cos\left(\phi_j'-\theta_r\right)\right)^2\right\}^{1/2} \tag{16}$$

$$= B_{1,b}\sqrt{1 + \left(\frac{\left|\vec{B}_{1,j}(r,\theta_r)\right|}{2B_{1,b}}\right)^2 + \frac{\left|\vec{B}_{1,j}(r,\theta_r)\right|}{B_{1,b}}\sin\left(\phi_j'-\theta_r\right)}. \tag{17}$$

$$\left|B_1^+(r,\theta_r)\right| = B_{1,b}\left\{1 + \left(\frac{\mu_0\mu_r I\cos\xi_j}{B_{1,b}4\pi r\left(1-\sin^2\xi_j\cos^2\left(\theta_r-\theta_j\right)\right)}\right)^2 - \frac{\mu_0\mu_r I\cos\xi_j}{B_{1,b}2\pi r\left(1-\sin^2\xi_j\cos^2\left(\theta_r-\theta_j\right)\right)}\sin\left(\theta_r-\phi_j'\right)\right\}^{\frac{1}{2}}. \tag{18}$$

$$\phi_{B_1^+}(r,\theta_r) = -\phi_b + \arg\left(\frac{2B_{1,b}}{\left|\vec{B}_{1,j}(r,\theta_r)\right|} + ie^{-i(\phi_j'-\theta_r)}\right) \tag{19}$$

$$= -\phi_b + \arctan\left(\frac{\frac{\left|\vec{B}_{1,j}(r,\theta_r)\right|}{B_{1,b}}\cos\left(\phi_j'-\theta_r\right)}{2+\frac{\left|\vec{B}_{1,j}(r,\theta_r)\right|}{B_{1,b}}\sin\left(\phi_j'-\theta_r\right)}\right). \tag{20}$$

So far, the explicit time-dependence noted in the equations above referred to the harmonic RF modulation of the fields. During a pulse sequence, amplitude-modulated RF pulses of varying peak $B_{1,b}$ are applied. The amplitude modulation of the RF pulses causes an identical modulation of the wire current over time, and thus of $B_{1,j}^+$. In turn, the total B_1^+ is subject to the same temporal modulation as $B_{1,b}$, the ratio between the two being constant in time. Equation 18 thus also holds for the respective RMS values of $B_{1,b}$ and I.

The assumption of a completely homogeneous background $B_{1,b}$-field in the entire sample is usually not realistic. This field can still be considered homogeneous over the region around the wire which is analyzed to fit the current, but the $B_{1,b}$ local to the wire may differ from the nominal value $B_{1,nom}$. Define $\lambda_b = \frac{B_{1,b}}{B_{1,nom}}$ to be the local B_1 heterogeneity of the coil-generated (background) transmit field, $\lambda_w\left(\vec{r}, \frac{I}{B_{1,b}}, \xi_j, \theta_j, \phi_j'\right) = \frac{B_1^+(\vec{r})}{B_{1,b}}$ the wire-generated B_1 scaling factor in Eq. 18 and $\lambda_{tot}(\vec{r}) = \frac{B_1^+(\vec{r})}{B_{1,nom}}$ the total B_1^+ heterogeneity including the effect of the wire current.

Lastly, it may be of interest to express the wire current I_{ref} with respect to a fixed reference $B_{1,ref}$ which does not depend on the pulse sequence. This would be the current observed with a sequence of $B_{1,nom} = B_{1,ref}$. The ratio $\frac{I(B_{1,nom},t)}{B_{1,nom}(t)} = \frac{I_{ref}}{B_{1,ref}}$, with the time-dependence here referring to the RF amplitude modulation during the sequence, is the same for all sequences and at all instants during the sequence, independently of their nominal RF amplitude. This value depends only on sample and machine properties such as transmit coils used, their respective transmit phase, path of the wire and sample properties. Taken together we can re-write the expression for the total transmit B_1-field in the presence of the wire as:

$$\left|B_1^+(\vec{r},t)\right| = B_{1,b}(t)\,\lambda_w\left(\vec{r}, \frac{I(t)}{B_{1,b}(t)}, \xi_j, \theta_j, \phi_j'\right) \tag{21}$$

$$\lambda_{tot}(\vec{r}) = \lambda_b\lambda_w\left(\vec{r}, \frac{I(t)}{\lambda_b B_{1,nom}(t)}, \xi_j, \theta_j, \phi_j'\right) \tag{22}$$

$$= \lambda_b\lambda_w\left(\vec{r}, \frac{I_{ref}}{\lambda_b B_{1,ref}}, \xi_j, \theta_j, \phi_j'\right). \tag{23}$$

Inserting the full expression for λ_w in Eq. 23 yields the final Eq. 24. The ratio of observed transmit field to nominal transmit field, λ_{tot}, can be calculated at each position $\vec{r}$ around the wire, given the current amplitude I_{ref} that would be observed for a fixed nominal reference field $B_{1,ref}$, as well as current phase and wire angulation. Since λ_{tot} does not depend on the nominal B_1 of the sequence, as expected (Maxwell equations are linear), it is identical to the ratio between the actually observed

local flip angle at position $\vec{r}$ and the nominal flip angle, for all flip angles of the sequence. Calculation of the da-hdrAFI signals is then straightforward, using for each voxel the actual local flip angle and the known signal equations. Given this forward model, it is possible to inversely fit the current I_{ref} from observed da-hdrAFI signals.

$$\lambda_{tot}(\vec{r}) = \lambda_b \left\{ 1 + \left(\frac{\mu_0 \mu_r I_{ref} \cos\xi_j}{\lambda_b B_{1,ref} 4\pi r \left(1 - \sin^2\xi_j \cos^2(\theta_r - \theta_j)\right)} \right)^2 - \frac{\mu_0 \mu_r I_{ref} \cos\xi_j}{\lambda_b B_{1,ref} 2\pi r \left(1 - \sin^2\xi_j \cos^2(\theta_r - \theta_j)\right)} \sin\left(\theta_r - \phi_j'\right) \right\}^{1/2} \tag{24}$$

2 | VALIDITY OF THE USE OF AMPÈRE'S LAW TO CALCULATE THE MAGNETIC FIELD AROUND A DBS LEAD WITH HELICAL INTERNAL STRUCTURE

Above, an analytical model of B_1^+ around a straight wire was derived based on the azimuthal $\vec{B}$-field generated by the axial RF current $I(t)$. In the case of realistic implants such as DBS leads, the internal structure is composed of multiple interlaced helical wires. The justification to use Equation 1 in this context is not straightforward. The use of this equation was previously justified in the context of MRI measurement of RF currents in implant wires based on the Ampère-Maxwell equation[1], which relates the line integral of the magnetic field $\vec{B}$ over a path C surrounding the lead or wire to a surface integral of the current density $\vec{j}$ over the surface S circumscribed by that path:

$$\oint_C \vec{B} \cdot d\vec{l} = \iint_S \left(\mu_0 \vec{j} + \mu_0 \varepsilon_0 \frac{\partial \vec{E}}{\partial t} \right) \cdot d\vec{S}. \tag{25}$$

In the context of a straight wire, the authors correctly argued that, in regions close to the wire, the high conductivity surface of the metal would impose a boundary condition which forces $\vec{E}$ to be perpendicular to $d\vec{S}$, allowing the second term to be neglected. It is not immediately clear that for a DBS lead this same argument holds, since it does not contain a continuous cylindrical metal surface. This is the reason we chose to validate the model based on full harmonic EM simulations of a realistic high-resolution model of a DBS lead. These simulations model the full 3D $\vec{B}$-field and a significant axial component of $\frac{\partial \vec{E}}{\partial t}$ would lead to an error in the RF current reconstructed from the simulated realistic B_1^+ when fitted with the simplified model.

Note that the first term on the right hand side of Equation 25 is not affected by the internal structure of the implant lead. Given that the implants considered do not contain any ferromagnetic material, the surface integral of the current density over any surface that entirely crosses the wire is identical to the total free current circulating inside the wire, or the sum of the currents of each wire if there is more than one wire as is frequently the case for DBS leads. This is independent of the angle between the surface considered and the lead and independent of the internal structure of the wire, be it straight or helical. Regarding the part of the B-field generated by the free current on the wire, we can therefore conclude that the use of Ampère's circuital law remains valid in determining the azimuthal magnetic field in the near field outside of such a lead. As noted in the manuscript, the axial and radial components of the RF $\vec{B}$-field generated by the azimuthal component of the current density in a helical wire have previously been shown to be of negligible amplitude outside of the wire[2].

3 | TESTED WIRE AND LEAD CONFIGURATIONS IN THE PHANTOM EXPERIMENT

Figure S1 shows T_1-weighted images of all tested configurations in the phantom experiments with the copper wire and the DBS electrode, annotated with the translations of the wire or lead tip and the respective angulations in case of the straight wire.

4 | RAW DATA OBTAINED IN THE PHANTOM EXPERIMENTS

Tables S1, S2 and S3 show the raw data obtained in the phantom experiment for all positions, respectively for the copper wire head side, copper wire feet side and DBS lead.

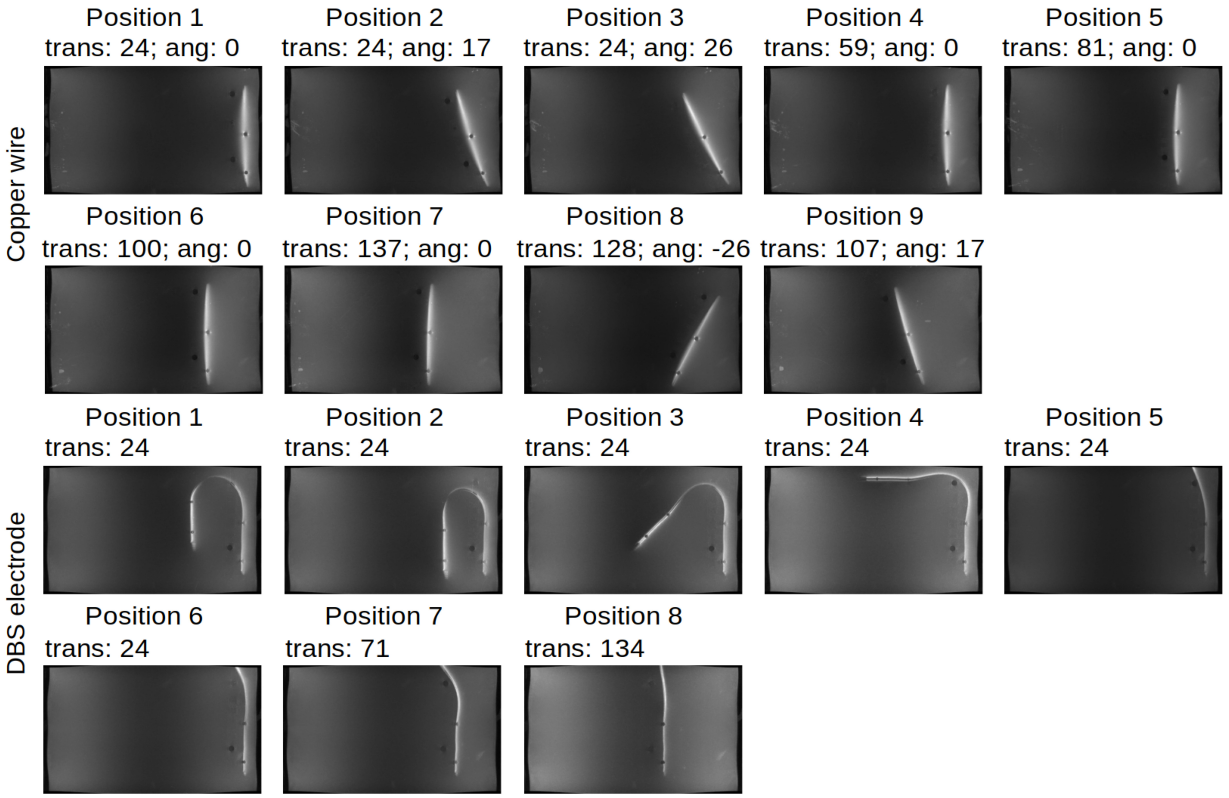

FIGURE S1 All tested configurations of the copper wire (9 positions) and DBS lead (8 positions). A 3D FLASH sequence is shown, 1-mm isotropic resolution, maximum intensity projection; trans: lateral translation of the wire tip (feet side) or DBS lead tip with respect to the lateral phantom wall (mm); ang: angulation of the straight wire (°).

TABLE S1 Data from the phantom experiment with copper wire ("head" side) for all positions of the wire. dT_{thermo}: corresponding temperature increase measured by temperature probe at the head-side wire tip during the heating sequence; dT_{MRI}: temperature increase predicted from the MRI current measurement; I_{thermo}: current derived from the temperature increase measured by temperature probe; I_{MRI}: RF current measured by MRI in a slice centered at 33 mm from the head-side wire tip; $dT\left(B_{1,rms}^{thresh}\right)$: temperature increases that would have been observed for a sequence at $B_{1,rms}^{thresh}$ derived from the MRI current measurement. All data correspond to a leave-one-out analysis as described in the manuscript.

**Position**	dT_{thermo} **(K)**	dT_{MRI} **(K)**	I_{thermo} **(A)**	I_{MRI} **(A)**	$dT\left(B_{1,rms}^{thresh}\right)$ **(K)**
1	16.0	18.4	0.330	0.354	1.74
2	15.0	14.0	0.328	0.317	2.15
3	11.7	11.1	0.288	0.281	2.10
4	14.3	12.6	0.321	0.302	2.26
5	11.5	11.4	0.286	0.284	2.02
6	8.1	7.8	0.239	0.234	2.08
7	3.6	3.6	0.161	0.159	2.04
8	6.4	7.7	0.212	0.232	1.67
9	3.2	3.3	0.151	0.152	1.96

TABLE S2 Data from the phantom experiment with copper wire ("feet" side) for all positions of the wire. dT_{thermo}: corresponding temperature increase measured by temperature probe at the feet-side wire tip during the heating sequence; dT_{MRI}: temperature increase predicted from the MRI current measurement; I_{thermo}: current derived from the temperature increase measured by temperature probe; I_{MRI}: RF current measured by MRI in a slice centered at 33 mm from the feet-side wire tip; $dT\left(B_{1,rms}^{thresh}\right)$: temperature increases that would have been observed for a sequence at $B_{1,rms}^{thresh}$ derived from the MRI current measurement. All data correspond to a leave-one-out analysis as described in the manuscript.

**Position**	dT_{thermo} **(K)**	dT_{MRI} **(K)**	I_{thermo} **(A)**	I_{MRI} **(A)**	$dT\left(B_{1,rms}^{thresh}\right)$ **(K)**
1	15.9	14.9	0.306	0.297	2.13
2	11.1	13.0	0.251	0.271	1.71
3	8.4	9.8	0.220	0.237	1.73
4	16.4	13.9	0.315	0.289	2.37
5	12.5	14.6	0.266	0.287	1.72
6	8.2	7.7	0.219	0.213	2.12
7	3.7	3.8	0.147	0.149	1.94
8	11.5	10.0	0.260	0.243	2.30
9	3.7	4.2	0.147	0.156	1.78

TABLE S3 Data from the phantom experiment with the DBS lead for all tested configurations. dT_{thermo}: corresponding temperature increase measured by temperature probe at the most distal DBS electrode contact during the heating sequence; dT_{MRI}: temperature increase predicted from the MRI current measurement; I_{thermo}: current derived from the temperature increase measured by temperature probe; I_{MRI}: RF current measured by MRI in a slice centered at 33 mm from the DBS lead tip; $dT\left(B_{1,rms}^{thresh}\right)$: temperature increases that would have been observed for a sequence at $B_{1,rms}^{thresh}$ derived from the MRI current measurement. All data correspond to a leave-one-out analysis as described in the manuscript.

**Position**	dT_{thermo} **(K)**	dT_{MRI} **(K)**	I_{thermo} **(A)**	I_{MRI} **(A)**	$dT\left(B_{1,rms}^{thresh}\right)$ **(K)**
1	7.0	5.9	0.163	0.150	2.37
2	7.4	7.2	0.165	0.163	2.06
3	5.1	5.0	0.136	0.135	2.03
4	4.0	4.7	0.120	0.129	1.73
5	2.5	3.3	0.095	0.109	1.50
6	2.2	2.8	0.090	0.100	1.60
7	1.6	1.7	0.075	0.078	1.87
8	1.1	0.7	0.062	0.049	3.29

REFERENCES

1. van den Bosch Michiel R., Moerland Marinus A., Lagendijk Jan J. W., Bartels Lambertus W., Berg Cornelis A. T.. New method to monitor RF safety in MRI-guided interventions based on RF induced image artefacts: Method to monitor RF safety in MRI-guided interventions. *Medical Physics*. 2010;37(2):814–821.
2. Tokaya J.P., Raaijmakers A.J.E., Luijten P.R., Bakker J.F., Van Den Berg C.A.T.. MRI-based transfer function determination for the assessment of implant safety. *Magnetic Resonance in Medicine*. 2017;78(6):2449–2459.
